# Clinicopathologic Features of Gastroenteropancreatic Neuroendocrine Tumors: A Single-center Experience

**DOI:** 10.4274/balkanmedj.galenos.2020.2020.1.126

**Published:** 2020-08-11

**Authors:** Tuğba Akın Telli, Ece Esin, Şuayib Yalçın

**Affiliations:** 1 Department of Medical Oncology, Hacettepe University Cancer Institute, Ankara, Turkey

**Keywords:** Carcinoid tumor, gastroenteropancreatic neuroendocrine tumor, neuroendocrine tumor

## Abstract

**Background:**

Gastroenteropancreatic neuroendocrine tumors, a heterogeneous group of neoplasms, originates from the neuroendocrine system of the gastrointestinal tract and pancreas. There are limited number of studies investigating neuroendocrine tumors in Turkey.

**Aims:**

To define the clinicopathologic, demographic, and survival features of patients with gastroenteropancreatic neuroendocrine tumors.

**Study Design:**

A retrospective observational cohort study.

**Methods:**

We reviewed hospital records of patients and data was analyzed retrospectively. We investigated the clinical, pathological, survival features, and prognosis of patients with gastroenteropancreatic neuroendocrine tumors (n=128) admitted to the medical oncology department between year 2003 and 2014. Survival estimation was performed by the Kaplan-Meier method. Univariate and multivariate Cox regression models were utilized to investigate the prognostic factors for survival.

**Results:**

Of 128 patients with gastroenteropancreatic neuroendocrine tumors, 61 (47.7%) were female and 67 (52.3%) were male. The most common site of the tumor was stomach (36.7%), while the most common stage of tumor at diagnosis was stage 4 (40.9%). The median follow-up period was 37 months, while the 3- and 5-year overall survival rates were 78% and 69%, respectively. The factors significantly affecting overall survival rate were clinical stage, grade, presence of metastasis at diagnosis, and Ki-67 proliferation index. These factors were associated with the 3- and 5-year overall survival rate. Moreover, grade (hazard ratio: 8.34, 95% confidence interval: 2.16-32.22, p=0.01) and presence of metastasis at diagnosis (hazard ratio: 3.18, 95% confidence interval: 1.30-7.77, p=0.01) independently predicted overall survival in multivariate model following adjustment for age and gender.

**Conclusion:**

Higher-grade and presence of metastasis at diagnosis are negative independent prognostic indicators of survival in patients with gastroenteropancreatic neuroendocrine tumors.

Gastroenteropancreatic neuroendocrine tumors (GEP-NETs) are rare tumors that exhibit heterogeneous biological, functional and clinical behaviors (1). Neuroendocrine cells arise from various tissues of the body. Previous studies have shown that jejunum/ileum and pancreas are the most common primary sites of GEP-NETs, with changing percentages between 16-29% and 31-34% in various demographic cohorts (2,3,4). There is a remarkable increase in the prevalence of GEP-NETs as a result of increased awareness of disease and advancement in diagnostic procedures.

Histological differentiation, grading, and staging of tumor tissue determine the method of treatment and prognosis of GEP-NETs (5,6). According to World Health Organization (WHO) 2010 classification, GEP-NETs are grouped into 3 as follows: well differentiated tumors separated into low-grade (G1) (mitotic count <2/10 high-power field (HPF) and/or Ki-67 index <3%) and intermediate-grade (G2) (mitotic count 2-20/10 HPF and/or Ki-67 index 3-20%), and poorly differentiated tumors as high-grade (G3) neuroendocrine carcinomas (mitotic count >20/10 HPF and/or Ki-67 index >20%) (7,8). However, recent studies have demonstrated that high-grade (G3) tumors may exhibit a heterogeneous biological behavior, affecting both prognosis and response to treatment (9,10).

This study aimed to define the clinicopathologic, demographic and survival features of patients with GEP-NETs diagnosed and treated in a tertiary reference oncology center for neuroendocrine tumors.

## MATERIALS AND METHODS

We reviewed the records of 128 GEP-NET patients admitted to the department of medical oncology between year 2003 and 2014. We obtained ethical approval for this study from the Ethics Committee (number: 16969557-1201). Demographic and clinicopathological data of the patients including age, gender, tumor location, embryological origin, presence of carcinoid syndrome, Ki-67 proliferation index, mitotic activity, presence of lymph node, and distant metastasis, surgical and medical history of GEP-NET, and long term survival rate were collected.

Tumor grading was determined by Ki-67 proliferation index and mitotic activity according to WHO histopathological classification (2010). Staging was done according to AJCC/UICC TNM classification (7^th^ Edition). Overall survival (OS) was defined as the time from diagnosis to death/last follow-up.

### Statistical analysis

Statistical analysis was performed with Statistical Package for Social Sciences (SPSS) version 16.5 for windows (SPSS 16.5 Inc., Chicago, II, USA). Categorical variables were expressed as frequency (percentage). Survival estimation was done by the Kaplan-Meier method. Univariate and multivariate Cox regression analysis were applied to ascertain the factors that predict OS. Confidence interval (CI) was set as 95%, and p value less than 0.05 was adopted as statistically significant.

## RESULTS

### Demographic and clinicopathologic characteristics of patients

One hundred and twenty-eight GEP-NET patients were included in this study. Of the 128 patients, 67 (52.3%) were male and 61 (47.7%) were female. The median age of the patients was 51.5 (range: 17-81). Exactly 5 (3.9%) patients had carcinoid syndrome, while 3 (2.3%) patients had MEN-1 syndrome. The most common sites of the tumor were stomach (36.7%) and pancreas (30.5%) followed by small bowel/appendix (15.6%), colon-rectum (7%), and metastatic NETs of unknown primary site (4.7%). Other sites include ampulla of vater (3.9%), liver (0.8%), and gall bladder (0.8%). Of the 47 patients with gastric NETs, type 1, 2 and 3 gastric NET distribution were as follows: type 1: 27 cases, type 2: 1 case, and type 3: 19 cases. Gastric NET type 1 was detected more in women (19 women vs 8 men), while type 3 gastric NET was detected more in men (13 men vs 6 women).

Ki-67 index ≤2% were detected in 40 (31.3%) patients, between 3% and 20% in 25 (19.5%) patients, and >20% in 14 (10.9%) patients. However, Ki-67 index was not detected in 38.3% of the patients. A total of 33 (25.8%) patients had mitotic rate of <2/10, 20 (15.6%) patients had mitotic rate of 2 to 20/10, and 2 (1.6%) patients had mitotic rate of >20/10. Grade of 17 tumors could not be classified, while over half of the tumors (56.8%) were grade 1, 28.8% were grade 2, 11.7% were grade 3, and 2.7% were evaluated as MANEC according to WHO 2010 classification.

Of 115 patients whose stages could be classified based on TNM, 16 (13.9%) patients were staged 0, 18 (15.7%) patients were staged 1, 15 (13%) patients were staged 2, 19 (16.5%) patients were staged 3, and 47 (40.9%) patients were staged 4. Overall, 47 (36.7%) patients had distant metastasis at diagnosis. The most common metastatic organ was liver, with 45 patients having liver metastasis, 3 patients having liver and bone metastasis, 2 patients having liver and ovarian metastasis, 1 patient having lung metastasis, and 1 patient having brain metastasis. Characteristics of the patients with GEP-NET are summarized in Table 1.

### Therapeutic interventions

Overall, 90 (70.3%) GEP-NET patients underwent surgery with curative intent. Endoscopic radical surgery including endoscopic mucosa resection and endoscopic submucosal dissection were performed in 11 patients with gastric NETs. Only 14 patients underwent metastasectomy for liver metastases. Locoregional therapies such as transarterial chemoembolization, radiofrequency ablation and transarterial radioembolization were carried out in 11, 6, and 12 patients, respectively. A total of 69 patients received systemic treatment including chemotherapy and biological therapy. Exactly 44 patients received somatostatin analogs, with half of them (22 patients) being first-line treatment. The most common first-line chemotherapy combinations included platinum-etoposide (24 patients) and streptozocin-based chemotherapy (11 patients). Also, 7 patients received everolimus and 1 patient received sunitinib. Eleven patients received capecitabine-temozolomide regimen as second-line or subsequent therapy.

### Survival and prognostic factors

**Figure 1:**
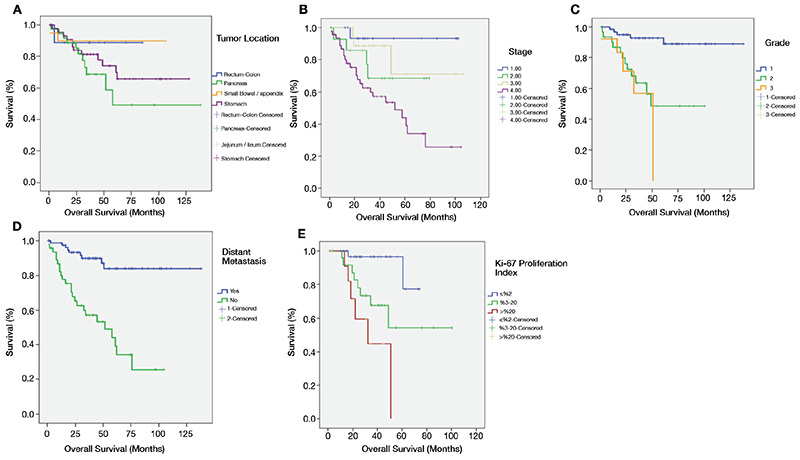
**A-E.** Overall survival (A), overall survival by tumor location (B), overall survival by stage (C), overall survival by histological grading (D), overall survival by presence of distant metastasis (E), and overall survival by Ki-67 proliferation index of GEP-NET patients. GEP-NET: gastroenteropancreatic neuroendocrine tumor

After a median follow-up of 37 months, the 3- and 5-year OS rate was 78% and 69%, respectively. Cancer-related deaths (specifically due to GEP-NETs) occurred in 33 (25.8%) patients. While median OS has not yet been reached for all patients, it was 50.9 months (95% CI: 21.5-80.3) for the metastatic category. Univariate analysis was done by age, gender, primary tumor site, embryological origin, histopathological grading, stage, Ki-67 proliferation index, and presence of metastasis at diagnosis in order to identify prognostic factors for survival. Stage (p=0.001), grade (p<0.001), presence of metastasis at diagnosis (p<0.001) and Ki-67 proliferation index (p=0.01) were found to be significantly related to OS. Clinical stage 3/4, higher grade, metastatic presentation, and higher Ki-67 proliferation index were significantly poor prognostic factors. On the other hand, age, gender, primary site of tumor, and embryological origin were not significantly related to survival rates in the Univariate analysis. The significant prognostic factors related to OS in Univariate analysis were subjected to multivariate analysis, with adjustment for age and gender. Grade (hazard ratio (HR): 8.34, 95% CI: 2.16-32.22, p=0.01) and presence of metastasis at diagnosis (HR: 3.18, 95% CI: 1.30-7.77, p=0.01) independently predicted OS. Higher grade and metastasis were identified as independent predictors of poor survival. There was no significant difference in 3- and 5-year OS rate between pancreatic NETs (PNET) and non-pancreatic NETs (p=0.316). Univariate and multivariate analysis of factors for predicting OS are summarized inTable 2, and survival curves are shown in Figure 1.

## DISCUSSION

GEP-NETs are heterogeneous neoplasms originating from diffuse endocrine system of gastrointestinal tract which may occur in different anatomic locations. It is logical to classify these tumors as PNETs and gastrointestinal NETs based on genetic and anatomic differences (11,12). These tumors may have different clinical presentations as a result of the release of endocrine secretions such as serotonin or histamine. Some PNETs are functional tumors that produce hormones which causes clinical syndromes. It is critical to manage symptoms associated with excessive hormone secretion to ensure a better quality of life and survival (13). Although these tumors have been considered as rare neoplasms, many studies have reported an increased incidence in recent years as a result of the advancement in diagnostic procedures (3).

Studies have shown that the small bowel and appendix are the most common sites for NETs (14,15,16). In our study, the most common tumor site was the stomach (36.7%) followed by pancreas (30.5%), small bowel/appendix (15.6%) and colon-rectum (7.0%). US Surveillance Epidemiology and End Results (SEER) national registry of cancer between year 1973 and 1997 were analyzed, and 11.427 patients were diagnosed with carcinoid tumors. Of the 11.427 cases, the most common tumor site was the small bowel (44.7%) followed by rectum (19.6%), appendix (16.7%), colon (10.6%) and stomach (7.2%) (16). In 2012, Wang et al. (17) reported in their retrospective analysis of 178 patients in a single-institution in South China that the most common site was the pancreas (34.8%) followed by rectum (20.2%) and stomach (14.0%). Recently, Yalcin et al. (18) reported a real-world data on diagnosis and treatment management in >1000 patients with GEP-NETs from 15 countries, and found that the most common tumor sites are the pancreas (43%) and stomach (17%). It is obvious that GEP-NET site location changes from center to center. The most logical explanation for these inconsistencies include ethnicity and racial disparities, geographic region of the center, as well as experience of the center.

According to the retrospective analysis of 71 patients who were followed up by Dogan et al. (19) at Ankara University Medical School between year 1997 and 2008, 53% of the patients were female and 47% were male. Also, Maggard et al. (16) reported 56% occurrence of GEP-NETs in female and 44% in male. In contrast, our study revealed that 47.7% of the patients were female and 52.3% were male. No significant difference was found in tumor localization based on sex. However, between the gastric NET subtypes, gastric NET type 1 was detected more in women (19 women vs 8 men), while type 3 was detected more in men (13 men vs 6 women). This may be explained by the fact that type 1 gastric carcinoids usually develop in patients with chronic atrophic gastritis, which is an autoimmune disease. Moreover, most autoimmune diseases are more prevalent in women than in men (20,21).

In SEER database, the mean age of the GEP-NET population was 61.4 years. While patients with appendix tumors were the youngest (54.4 years), patients with small bowel tumors were the oldest (65.1 years) (16). In our study, the mean age was 50.45 years, and there was no significant difference in age at the time of diagnosis between the groups (p=0.429). The 10-year gap between the median diagnostic ages of NETs in two studies was determined mainly by our younger population. In general, for most malignancies, the median age of diagnosis is within the young age range when compared to world cancer statistics. In addition, patients included in our study were selected between year 2003 and 2014. Further, the advancement in technology and diagnosis methods in the past 2 decades could be another reason for the diagnosis of NETs at earlier age.

In 2003, Modlin et al. (14) published their findings on 13715 carcinoid tumors cases. They reported a distant metastasis rate (DMR) of 25.7% between year 1973 and 1991, and 15.5% between year 1992 and 1999. Also, Wang et al. (17) reported a DMR of 23% at diagnosis, and 28.1% during follow-up. In our study, de-novo metastatic patients accounted for 36.7% of all cases. In terms of primary site, 46.2% of pancreatic NETs, 25.5% of gastric NETs and 41.2% of small bowel NETs were metastatic at the time of diagnosis. The hypothesis of late diagnosis of pancreatic NETs is due to its asymptomatic presentation. Gastric NETs that were metastatic at diagnosis were type 3, which are regarded as already aggressive neuroendocrine tumors, although histological parameters show intermediate proliferative indices. There may be several reasons for the higher de-novo metastatic rate in this study. Firstly, only 5 out of 128 patients (3.9%) had carcinoid syndrome, indicating that the patients were admitted into the hospital in late stages due to the asymptomatic nature of disease and lack of awareness. Secondly, our clinic is the referral oncology center for NETs, and while metastatic patients are directed to our center, patients with early stages of disease are treated locally. In our study, the most common metastatic organ for GEP-NETs was the liver. Other metastatic sites were bone, ovary, lung, and brain.

As surgery is the only potential curative treatment option for GEP-NETs, it should be considered both in the early stages and resectable metastatic stage of the disease (22). In our study, 70.3% of the patients underwent surgery with a curative intent. Of 111 patients with classified tumors, 56.8% were grade 1, 28.8% were grade 2 and 11.7% were grade 3. Wang et al. (17) reported the rate of curative intent surgery as 75.9%, with 51.5% of these patients evaluated as grade 1, 18.3% as grade 2 and 30.2% as grade 3. Additionally, Foltyn et al. (23) evaluated the prognostic role of Ki-67 proliferation index in 2012. Of the 61 patients included in the study, 62.3% were grade 1, 19.7% were grade 2 and 18% were grade 3.

In our study, median follow-up time was 37 months, and the 3- and 5-year OS rates were 78% and 69%, respectively. In the Univariate analysis, small intestine-appendix, and colorectal NETs were demonstrated to have the best prognosis, with the 3- and 5-year OS rate of 90% and 89%, respectively. While the 3- and 5-year OS rates for pancreatic NETs were 69% and 49%, respectively, it was 81% and 74%, respectively, for gastric NETs. There was no difference in the 3- and 5-year OS rates between the groups based on the tumor site of origin (p=0.275). Pancreatic and gastric NETs were diagnosed at late stages with higher grades, therefore, their OS rates were least favorable.

In a study by Modlin et al. (14) on 13715 patients with carcinoid tumors diagnosed between year 1992 and 1999, the 5-year OS rate was 67.2%. Further, according to the localization of the tumor, the best results were obtained in rectal NETs (5-year OS rate: 87.5%) followed by appendix and small intestine, with 5-year OS rate of 76.3% and 76.1%, respectively. In their study, 5-year OS rate was 75.1%, with the worst result obtained in colon NETs (5-year OS rate: 69.5%). In the study described above, factors affecting OS were noted as tumor stage and presence of metastasis. Foltyn et al. (23) concluded that Ki-67 proliferation index is an important and necessary parameter for the prognosis of GEP-NETs. In a study by Van Gompel et al. (15) published in 2004, it was found that the most important factors affecting OS were embryological origin and symptomatic presentation, whereas the size of primary tumor and presence of liver metastasis did not predict survival. Wang et al. (17) showed that the 3- and 5-year OS rates were 66.7% and 54.5%, respectively. In their study, it was concluded that the most important factors associated with OS were grade, functional status, and presence of distant metastasis. In study by Yucel et al. (24) with 52 cases published in 2013, the 3-year OS rate was 71%. In the subgroup analysis, OS rate was 100% in stage 1, 88% in stage 2, 80% in stage 3 and 40% in stage 4. In their study, gender, age, performance status, grade, tumor localization, surgical treatment and neutrophil/lymphocyte ratio (≤5 or >5) were found to affect prognosis, however, only three of them were independent prognostic factors; which are surgical treatment (HR: 0.003, 95% CI: 0.006-0.159, p<0.001), tumor of grade 3 (HR: 11.8, 95% CI: 1.9-72.8, p=0.007) and a neutrophil/lymphocyte ratio of >5 (HR: 4.4, 95% CI: 1.2-15.7, p=0.022). On the other hand, in study by Esin et al. (25) with 72 patients of well differentiated NETs, the 5-year OS rate was 77.5%, and there were no relationships between grade, Ki-67 proliferative index, and OS rates. In another study by Yildiz et al. (26) on a retrospective data of 86 patients with GEP-NETs, the factors significantly correlated with survival were number of lymph nodes, multifocality, metastases, and stage; however, no independent variable was determined in multivariate analysis.

Since many studies have reported various parameters as prognostic factors for OS in patients with GEP-NETs, we performed our statistical analysis based on all abovementioned demographic, clinical, and pathological determinants. Based on the Univariate analysis, the most important factors affecting OS significantly were clinical stage, grade, presence of distant metastasis at diagnosis and Ki-67 proliferation index. Among these factors, higher grade (HR: 8.34, 95% CI: 2.16-32.22, p=0.01) and metastatic presentation (HR: 3.18, 95% CI: 1.30-7.77, p=0.01) were independent predictors of poor survival in the multivariate model.

Our study has some limitations. Firstly, a relatively low number of sample size, especially in the categories of variables, may negatively affect the statistical analysis. However, a post-hoc power analysis revealed a power >0.9 in most of the survival analysis, indicating that the number of subjects in the current study was adequate for the statistical analysis. Secondly, this study contains some of the inherent biases of retrospective study designs. Thirdly, majority of the patients had advanced stage NET, making our cohort heterogeneous. However, our survival analysis was successful when compared to the best data reported so far. Lastly, tumor grading was determined based on the previous WHO histopathological classification published in 2010.

In conclusion, this is a retrospective study of 128 GEP-NET patients who were diagnosed and/or treated in a reference cancer clinic. We demonstrated that higher grade and presence of metastasis at diagnosis are two negative independent indicators for survival in patients with GEP-NETs. Owing to the rarity of this tumor type and lack of necessary awareness among clinicians about NETs, further demographic NET registries are required to investigate the biology and course of the disease.

## Figures and Tables

**Table 1:**
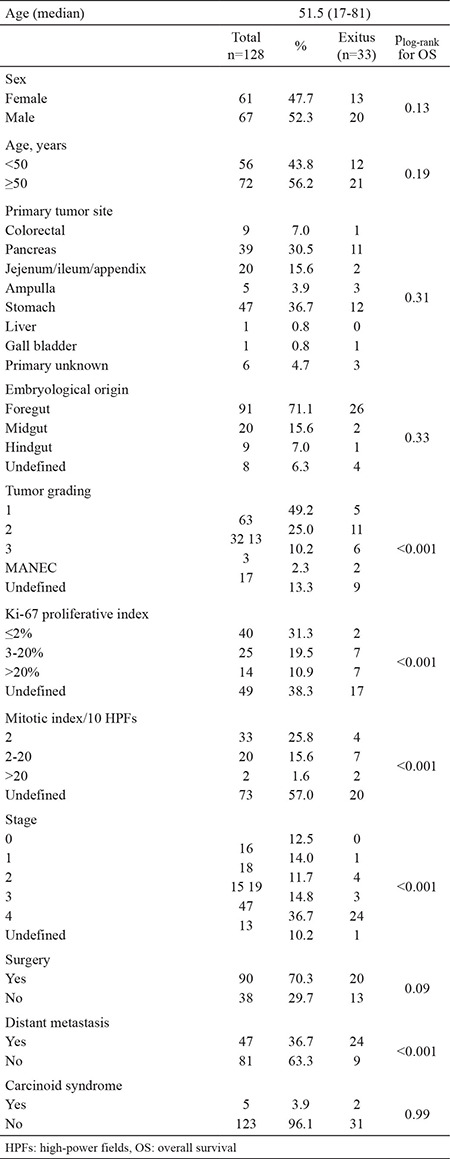
Characteristics of study population

**Table 2:**
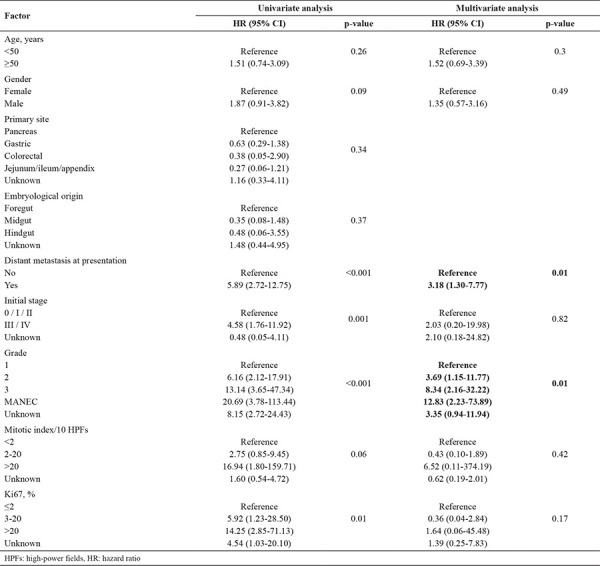
Univariate and multivariate analyses of factors for predicting overall survival
